# Quantification of specific bindings of biomolecules by magnetorelaxometry

**DOI:** 10.1186/1477-3155-6-4

**Published:** 2008-03-11

**Authors:** Dietmar Eberbeck, Christian Bergemann, Frank Wiekhorst, Uwe Steinhoff, Lutz Trahms

**Affiliations:** 1 Physikalisch-Technische Bundesanstalt, Abbestrasse 2-12, D-10587, Berlin, Germany; 2 Chemicell GmbH, Eresburgstrasse 22-23, D-12103, Berlin, Germany

## Abstract

The binding reaction of the biomolecules streptavidin and anti-biotin antibody, both labelled by magnetic nanoparticles (MNP), to biotin coated on agarose beads, was quantified by magnetorelaxometry (MRX). Highly sensitive SQUID-based MRX revealed the immobilization of the MNP caused by the biotin-streptavidin coupling. We found that about 85% of streptavidin-functionalised MNP bound specifically to biotin-agarose beads. On the other hand only 20% of antibiotin-antibody functionalised MNP were specifically bound. Variation of the suspension medium revealed in comparison to phosphate buffer with 0.1% bovine serum albumin a slight change of the binding behaviour in human serum, probably due to the presence of *functioning *(non heated) serum proteins. Furthermore, in human serum an additional non-specific binding occurs, being independent from the serum protein functionality.

The presented homogeneous bead based assay is applicable in simple, uncoated vials and it enables the assessment of the binding kinetics in a volume without liquid flow. The estimated association rate constant for the MNP-labelled streptavidin is by about two orders of magnitude smaller than the value reported for free streptavidin. This is probably due to the relatively large size of the magnetic markers which reduces the diffusion of streptavidin. Furthermore, long time non-exponential kinetics were observed and interpreted as agglutination of the agarose beads.

## Background

The binding reaction between different biomolecules, e.g. antibody-protein or ligand-receptor coupling, is of great interest in traditional and in modern fields of biosciences, e.g. proteomics. For example, the kinetics of association and dissociation reactions enables the estimation of the affinity of biomolecules. This is useful for studies on drug efficiency or therapeutic drug monitoring [1].

The detection and quantification of antigenes, e.g. specific surface proteins of bacteria or malignant cells, or specific extraneous biomolecules, is performed by so-called immunoassays. In immunoassays, detection molecules, e.g. antibodies, bind specifically to the analyte to be quantified. Signal transducers which are linked to the detection molecules give a physically measurable signal.

In heterogeneous immunoassays, the unbound markers have to be washed out in order to get a signal from bound marked detection molecules, only. In homogeneous immunoassays the signal of the transducers changes as the result of the binding of the detection molecule, i.e. the amount of bound detection molecules can be measured in the presence of unbound ones by separating the two qualitatively different signals. Besides their high potential for automation, homogeneous immunoassays enable the measurement of the binding kinetics [2]. Well-known examples of homogeneous assays are the Fluorescence Polarisation Immunoassay (FPIA) [3] and the Surface Plasmon Resonance (SPR) assay [4,5]. In the case of FPIA the signal transducers are fluorophores changing the polarisation of the emitted light after a binding. In the SPR based assay, the detection molecules are attached to a solid surface acting as anchors for the analyte molecules. The binding increases the mass attached to the sensor surface leading to a change of the surface plasmon resonance frequency.

Another homogeneous immunoassay is the MAgnetic Relaxation ImmunoAssay (MARIA) [6,7], where the detection molecules are labelled by magnetic nanoparticles acting as signal transducers.

In this paper we investigate the quantification of the biotin-streptavidin and biotin-antibiotin-antibody bindings and its kinetics using MARIA. Biotin serves as the analyte and streptavidin and antibiotin-antibody have the function of detection molecules.

In MARIA, the detection molecules are labelled by Magnetic NanoParticles (MNP). These composites are the probes for the analyte molecules. The relaxation of the magnetic moment of the MNP after switching off an external field by their relaxation behavior is measured by MagnetoRelaXometry (MRX) [8]. The relaxation signal depends on the binding state of the probes [6]. MARIA provides the quantity of immobilised probes, i.e. the amount of bound biomolecules, in the presence of the unbound ones because these two signal contributions can be separated. Consequently, MARIA is a homogeneous immunoassay which needs no washing steps and the kinetics of the binding is easily accessible. Because the magnetic signal is not influenced by non-magnetic components of biological material, the binding behavior of an analyte can be investigated in different environments (e.g. water, serum or blood) with only one method by MARIA.

MARIA does not require a special geometry, so that standard vials can be used. Thus, it is possible to quantify biomolecules in a realistic environment, without severe sample alterations due to preparation.

**Figure 1:**
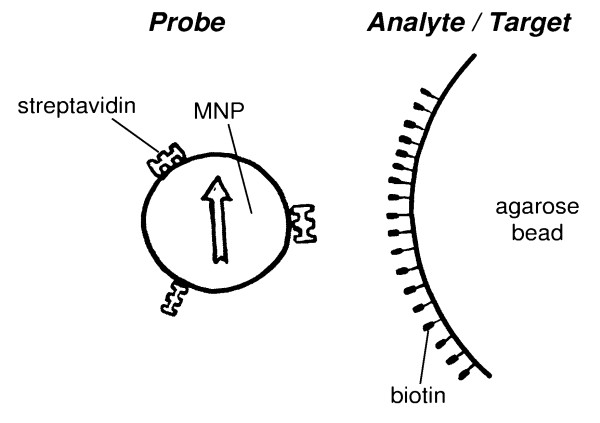
Relaxation curves of the MNP*SAV measured 15 min, 1 h, 5 h and 24 h after mixing with biotin-agarose. The relaxation curves of the control samples (see text) are also depicted. The relaxation curve of immobilized MNP (freeze-dried) is the reference for completely bound MNP.

MARIA was already used for *in-vitro *applications, e.g. the quantification of human IgG [9] or *Listeria monocytogenes *[10]. While most early studies [9] utilised a solid phase immunoassay, we here present the method of a bead based assay, where the analyte is coupled to *μ*m-sized beads (agarose beads) (Fig. 1). Thus, specially prepared reaction vials (e.g. coating with catching antibodies) are not necessary. Further, the measurement range can be enhanced because the total reaction surface (bead surface) can exceed that of the well surface, considerably. In contrast to the solid phase assay, here both components are mobile. This has advantages, for instance the distribution of the reactants is much more homogeneous. Also, the reaction kinetics should be faster and more clearly described than in a solid phase assay, due to less influence of mass transport phenomena on the binding kinetics. On the other hand, the present system has more degrees of freedom on the microscopic scale making it more complex. It arises the problem of a possible agglutination of the beads driven by the binding. In order to check the integrity of the method, we address the problem whether this enhanced complexity influences the feasibility and the range of linearity of this kind of assay. We investigated how the relatively large size of the marker affects the binding efficiency and the binding kinetics. Further, we examined the influence of the medium on the binding reaction, comparing the quantity of bound probes in 0.1% bovine serum albumin and human serum.

## Results and Discussion

### Quantification of the binding

**Figure 2:**
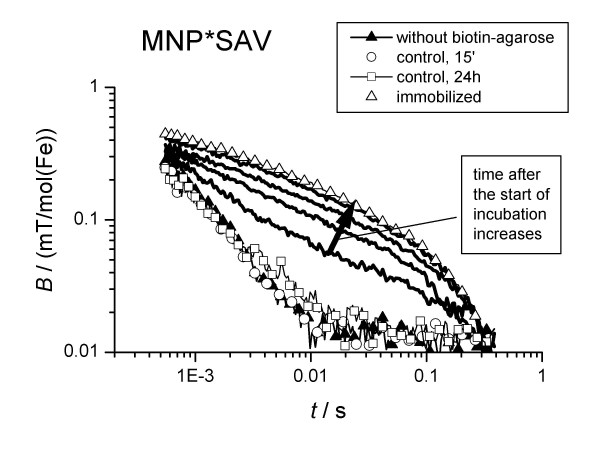
Relaxation curves of MNP*AB measured 15 min, 1 h, 5 h and 24 h after mixing with biotin-agarose.

**Figure 3:**
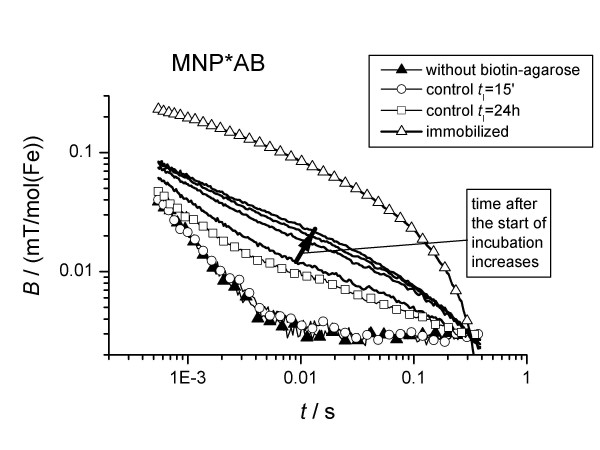
Influence of the MNP*SAV concentration on the binding kinetics. Samples were slowly rotated during incubation. The fraction of bound MNP*SAV as a function of incubation time is depicted for different MNP-dilution ratios. Solid curves represent the best fits according to (16).

First, aqueous suspensions of the magnetic probes alone were measured to provide a reference for the evaluation of relaxation changes due to the binding of the particles. In the absence of a binding target, the observed relaxation time of the sample moment, *τ*_obs_, was in the range of 2 ms. After incubation with biotin-agarose beads the relaxation time increased to up to 15 ms (Figs 2, 3), indicating the immobilisation of the MNP by the binding of the detection molecules to the large targets.

**Figure 4:**
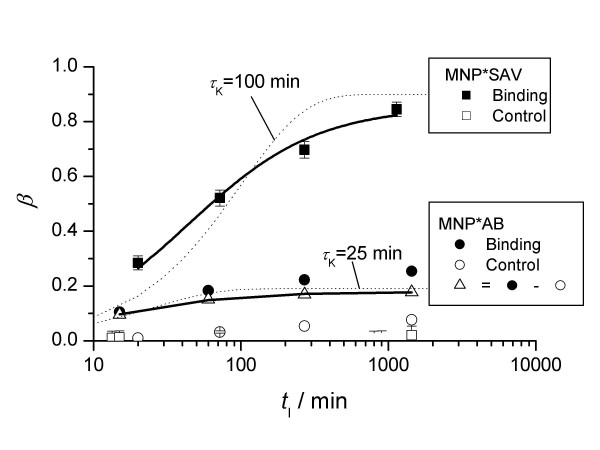
Fraction of bound MNP*SAV and MNP*AB as a function of incubation time. Subtraction of the amount of non-specific bound MNP*AB (control) yields the time dependence of its specific binding (open triangles). The solid lines are curves fitted according to (16). Exponential growth functions *β*(*t*_I_) = *β*_max _(1 - exp{-*t*_I_/*τ*}) (dotted lines) are fitted to show the deviation from exponential behavior.

**Figure 5:**
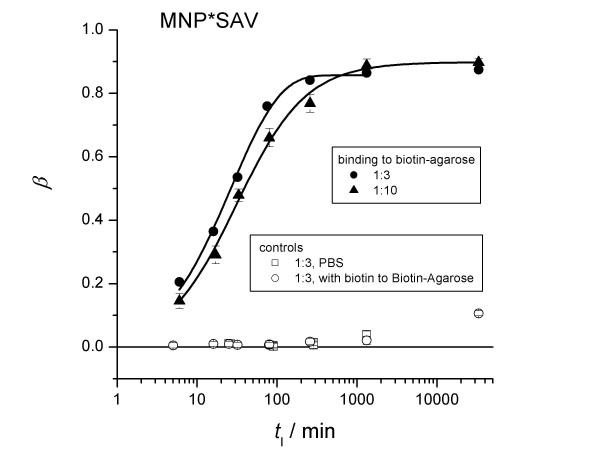
Relaxation curves for MNP*SAV2 binding to biotin-agarose in different suspension media. The incubation time is tI = 200'. In the control sample the SAV2-molecules of the MNP*SAV2 were saturated with free biotin before incubation with analyte suspension.

By fitting eq (2) to the measured relaxation curves, the amount of bound probes MNP*SAV (streptavidin linked to MNP), *β*, was calculated. The uncertainty is about 5%. With elapsing incubation time, *t*_I_, *β *increased (Fig. 4) to a level of 85%. We obtained similar results even at a 3 times higher concentration of MNP*SAV (Fig. 5). Thus, we cannot attribute the presence of 15% unbound MNP*SAV to a lack of biotin targets, but rather to the signal from idle MNP labels, e.g., to MNP carrying streptavidin in an inoperative configuration or to the presence of MNP which do not carry streptavidin-molecules at all.

On the other hand, in the control sample, where MNP*SAV saturated with biotin was incubated with biotin agarose, the same relaxation signal as that of the reference sample of MNP*SAV without the biotin agarose target (Fig. 2) was obtained. This indicates the absence of unspecific binding, so that the binding between MNP*SAV and agarose can be attributed exclusively to the specific streptavidin-biotin coupling.

The binding behaviour of MNP*AB (antibiotin-antibody linked MNP) to biotin agarose was quantitatively different. Even after one day of incubation, only 25% of the MNP*AB were coupled to the targets (Fig. 4). We obtained the same result in the case of a 10 times lower concentration of MNP*AB. Apparently, 75% of the labels are not immobilized, even in the excess of target molecules. One explanation might be again that there are MNP-labels without properly coupled detection molecules. Obviously, in the case of MNP*AB the fraction of idle labels is much larger than in the case of MNP*SAV.

The control experiment for MNP*AB again exhibits a behaviour that is different from MNP*SAV. After incubation with a high excess of free biotin, part of MNP*AB are immobilized in the presence of biotin agarose, indicated by an increased relaxation time, while no change in relaxation was observed for the samples without biotin agarose (Fig. 4). To explain this behaviour we propose two hypotheses: (i) The MNP*AB bind to the agarose-beads non-specifically. (ii) The antibiotin antibodies bind readily to biotin being part of larger compartments, e.g. biotinylated proteins, but only modestly to free biotin [11]. This causes the biotin, immobilised to agarose beads, to exchange the free biotin at the anti-biotin antibody.

### Binding kinetics

The continuous measurement of the magnetic relaxation during the incubation time *t*_I _provides information about the binding kinetics. By fitting equation (16) to the relaxation curves, we obtain *β*(*t*_I_) the actual relative amount of bound MNP. From our data the binding time *t*_b_, which is defined as the time at which *β*(*t*_b_) = (1/(1 - 1/*e*)) *β*_max_, where *β*_max _is the amount of bound probes for *t *→ ∞ in the saturation condition, was estimated to be *t*_b _= (9400 ± 1000) s and *t*_b _= (3600 ± 350) s for MNP*SAV and MNP*AB, respectively.

In these binding experiments (Figs 2, 3) the samples were shaken shortly just before each measurement sequence in order to homogenise the samples. Between the subsequent relaxation measurements that monitored the kinetics of the binding reaction the samples were in rest. In a second set of experiments the samples were rotated slowly between each relaxation measurement. With sample rotation, the binding kinetics was found to be faster with *t*_b _= (4400 ± 220) s and *t*_b _= (807 ± 100) s for MNP*SAV and MNP*AB, respectively (Table 1). Shaking and rotating should prohibit the sedimentation of agarose beads in the sample, which may hamper the diffusion of MNP*SAV to the biotin targets. In this way, the observed acceleration of the binding kinetics can be understood.

**Table 1:** Values of binding time estimated from the binding curves *β*(*t*_I_) as well as the association rate constants calculated according (11), (12) and the association rate constant for free streptavidin, take from [13].

			Association rate constant		
Samples	MNP-concentration *c*_P _(nmol/1)	binding time *t*_b _(s)	eq. (11) *k*_a _$\left(10^{3}\frac{\text{l}}{\text{mol s}}\right)$	eq. (12) *k*_a _$\left(10^{3}\frac{\text{l}}{\text{mol s}}\right)$	Ref. [13] *k*_a _$\left(10^{3}\frac{\text{l}}{\text{mol s}}\right)$
without agitation					
MNP*SAV	2	9400 ± 1000	54 ± 9	270 ± 45	5000
MNP*AB	23	3600 ± 350	12 ± 2	60 ± 10	
with agitation					
MNP*SAV	2	4400 ± 220	116 ± 20	580 ± 100	5000
MNP*SAV	6	2700 ± 60	63 ± 2	315 ± 10	5000
MNP*AB	23	807 ± 100	55 ± 10	275 ± 50	

As discussed in methodical section, we can calculate the association rate constants according to (11), which are listed in Table 1. With the obtained association rate constants equation 11 holds for *c*_P,0 _≫ *k*_d_/*k*_a,p _= 10^-12 ^$\frac{\text{mol}}{\text{l}}$, using *k*_d _≈ 10^-8 ^s^-1 ^[12,13]. This condition is fulfilled in all our experiments (Table 1).

In order to compare these values with those found for unlabelled streptavidin using SPR, we have first estimated the values for unlabelled streptavidin using (12). Assuming that the streptavidin molecules (SAV) have a "diameter" of about 10 nm we get a size ratio *κ *= *r*_P_/*r*_SAV _= 5 (mean diameter of the MNP*SAV is approximately 50 nm). Accordingly, the association rate constant for bare streptavidin becomes 3...6·10^5 ^$\frac{\text{l}}{\text{mol s}}$ depending on the agitation of the sample (Table 1). These values are one order below the reported association rate constants for streptavidin 5·10^6 ^$\frac{\text{l}}{\text{mol s}}$[13] and avidin 10·10^6 ^$\frac{\text{l}}{\text{mol s}}$[12]. Probably, this discrepancy is connected with the strong decrease of rotational diffusion (∝ $r_{\text{hyd}}^{-3}$) of SAV due to its association with MNP, which is not taken into account in (12). Furthermore, there is an agglutination of the agarose beads which may cause a reduction of the binding kinetics.

### Agglutination

The kinetics data *β*(*t*_I_) were well described by fitting a binding model (14) developed earlier for description the binding of charged MNP to oppositely charged latex spheres [14] (see appendix) (Fig. 4 and 5). However, there is a strong deviation from the expected exponential behavior (equation 10), represented by the dotted lines, especially for the non-agitated samples with the MNP*SAV probes (Fig. 4). Further, the obtained parameters seems to be far from the reality. For instance, the parameter *α*_F_, describing the fraction of binding active surface area of the agarose beads is only in the order of several percent.

We attribute this behavior to the agglutination of the agarose beads due to the binding of probes. This view is supported by the fact that agarose aggregates are visible with the eye after the binding reaction. This agglutination may also be responsible for the circumstance that the association rate "constant" depends on the concentration (Table 1).

Although the association constant of antibiotin-antibody to biotin *K*_a _= 10^9 ^$\frac{\text{l}}{\text{mol}}$[15] is much smaller than that of streptavidin (*K*_a _≈ 10^15 ^$\frac{\text{l}}{\text{mol}}$[16]) and although the numbers of antibiotin-antibodies per probe seems to be less than that for streptavidin probes (see above), the kinetics of MNP*AB-biotin-agarose coupling is in the same range like that of MNP*SAV-biotin-agarose coupling (Table 1). We attribute this behavior to a lower degree of agglutination of biotin-agarose beads by MNP*AB probes, confirmed by visual observation of the aggregates, which are stronger developed in the MNP*SAV samples than in the MNP*AB samples. This we explain as follows: Only about 20% of the MNP*AB probes bind specifically (see above). Therefore, we guess that the fraction of MNP to which more than 1 antibody is attached in the right direction, is much smaller than in the case of MNP*SAV. Because solely these MNP having more than one detection molecule can provoke cross-linking among the agarose beads, there is a lower probability of agglutination of agarose beads by MNP*AB probes than by MNP*SAV probes. If agglutination takes place, many binding places becomes hidden quickly after start of the incubation. Thus, a slowing down of the binding kinetics accompanied by a non-exponential behavior can be understood.

### Influence of the suspension medium

In this section we compare the binding of MNP-probes suspended in different media, namely BSA-Buffer (BSA-B), being PBS (phosphat buffered saline) with 0.1% BSA (bovine serum albumin), and human serum. Because we know that serum components can lead to additional agglutination effects in MNP-samples, probably due to active antibodies [17], we used also tempered serum. The serum was tempered at 60°C for 40 minutes leading to cancelling of this reason for agglutination [17].

**Figure 6:**
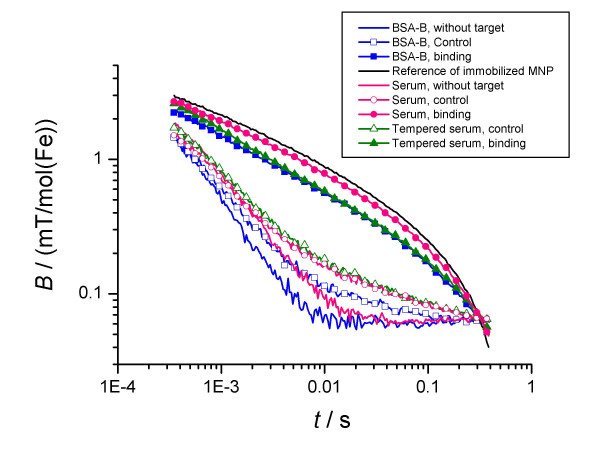
Sketch of the specific binding between streptavidin functionalised MNP and biotin-agarose. The magnetic nanoparticles (the arrow symbolises its moment) have a physical extent mean diameter of *d *≈ 50 nm and the diameter of biotin-agarose beads is (*d*_A _≈ 5 *μ*m). The large extent of streptavidin (*m*_w _= 53000 g/mol) is about 10 nm, whereas the biotin molecule is much smaller (*m*_w _= 244 g/mol).

First, we checked the influence of the suspension medium on the relaxation signal of the MNP-probes alone. The relaxation curve for MNP suspended in BSA-buffer is very similar in shape to that of human serum (Figure 6). But the relaxation time is by a factor of 1.4 smaller than that of the serum sample. This difference is related to the viscosity of serum of about 2 mPa s being twice of that of water: Analysing the relaxation curves with a model describing the hydrodynamic properties of the MNP [18] using the mentioned values for the dynamic viscosity, we get mean hydrodynamic diameters (diameter of objects with mean volume) of the relaxing entities (MNP and small clusters) of 76(2) nm, 65(2) nm and 76(2) nm for MNP suspended in BSA, human serum and tempered human serum, respectively. As argued above, the serum components obviously are responsible for a slight change in the MNP-aggregation in the suspension, represented by a change of the distribution of hydrodynamic sizes.

Here, it was demonstrated that the relaxation behavior of MNP can be measured in different media, what was already done in [18]. The data show, that the same results were obtained if the differences in the viscosity were taken into account.

Next, we discuss the binding behaviour in the different media. By fitting model (2) to the relaxation curves (Figure 5), we get the fraction of bound probes, *β *(Table 2). The results show clearly that there is an additional binding yield of 26% in the serum sample in comparison to the BSA-buffer based one. This difference disappears, if the serum was tempered before use. Therefore, we conclude that the activity of some serum proteins is responsible for this additional binding.

**Table 2:** Fraction of bound MNP*SAV2 to biotin-agarose beads after an incubation time *t*_I _in different suspension media estimated by fitting the MRX-curves by (2). The control sample means that SAV-molecules of the MNP*SAV2 are saturated with free biotin before incubation with analyte suspension.

		Fraction of bound MNP		
			Agarose beads	
Medium	*t*_I _min	no analyte	Control	Binding
BSA-B	200	0.018 ± 0.006	0.054 ± 0.007	0.61 ± 0.04
serum tempered	200	0.005 ± 0.008	0.117 ± 0.012	0.61 ± 0.04
serum	200	0.011 ± 0.008	0.114 ± 0.009	0.87 ± 0.06

The results obtained from the control samples show, that about 5% and 11% of the probes bind non-specifically in BSA-B and human serum, respectively. Because MNP*SAV shows no non-specific binding (Figure 1), we attribute the occurrence of the nonspecific binding in BSA-B to the changed batch of MNP-probes MNP*SAV2. However, serum components are obviously responsible for the enhancement of this non-specific binding of the MNP*SAV2 probes by about 6%. Note, that the MRX-curves of the control samples for serum and tempered serum are nearly identical. This means, that the non-specific binding is not related to the functioning of the serum proteins.

## Conclusion

We presented a method for quantification of biomolecules in the form of a bead based magnetic relaxation immunoassay. In a conventional solid phase immunoassay the analyte is fixed on the surface of the reaction well. The area of this active surface of one well (96 well plate) is 1.5·10^-4 ^m^2^. In our realised bead based assay the target surface area is about 1.5·10^-3 ^m^2 ^per well. The agarose beads and thus the target surface is more homogeneously distributed in the sample volume than in a solid phase system, where the analyte has to reach a surface coated with the detection molecules. Thus, in principle, in MARIA measurements in arbitrarily shaped volumes are possible.

Applying this method, we could quantify the binding of magnetically labelled streptavidin having a concentration of about 2 nmol/l with an accuracy of about 5% using a highly sensitive SQUID-measurement system. Given sensitivity was achieved by fitting a relaxation model to the relaxation data, whereby we could use the data of samples with fully immobilised MNP as a reference. By means of a control experiment we have shown that the probes were immobilised by specific streptavidin-biotin coupling or predominantly specific antibiotin-antibody-biotin coupling.

In the present assay design the analyte had to fixed to a relatively large bead enabling the detection by MRX. In order to quantify free analytes, the design has to be realised as a competition assay. I.e. the free analyte molecules compete with the bead based ones for the MNP-labelled detection molecules.

In the presented bead based binding assay the binding kinetics depends on the sample treatment during the incubation. In order to achieve reproducible kinetics, it is necessary to provide a controlled smooth sample stirring which prevents sedimentation of the larger beads.

Further, the size of the probes, i.e. MNP-detection molecule complexes, crucially determines the diffusion and hence the association rate constant. Presumably, the binding reaction is accompanied by agglutination processes affecting the binding kinetics. In order to estimate proper kinetics parameters binding experiments with an excess of probes have to be realised, suppressing the agglutination of agarose beads.

Obviously, the use of the large MNP as markers for biomolecules makes the estimation of the kinetics of the binding reaction difficult. However, the MRX method is suitable for a quantitative check of the functionalisation of MNP prepared for specific binding to an analyte. This is an important issue, because the research on systems of nanoparticles for diagnostic and therapeutical purposes is strongly increasing. For instance in tumor therapy and diagnostics (MRI) with MNP the focus tends from simple core shell MNP to functionalised (e.g. with an antibody) MNP which should find their target by specific binding [19]. Therefore, the complex binding behavior which can be accompanied by nonspecific binding has to be measured in order to develop such particles or in order to check its quality by the users.

## Methods

The bead based magnetic relaxation binding assay presented here (Fig. 1) has two peculiarities: (i) MNP that label the detection molecules have an overall diameter ranging from 15 nm to 60 nm or even more, i.e. in most cases the size of the marker is of the order or even larger than the size of the detection molecules. (ii) The analyte molecules are bound to beads being large in comparison to the markers. These two peculiarities make the signals of bound and unbound markers separable with high sensitivity. Furthermore, free analytes can be quantified by competition assay, i.e. the free analytes compete with the bead-associated ones for the binding sites of the probes.

### Quantification of the binding by MRX

The measurement is performed using a procedure and a device described in detail by Matz et al [20]. In short, a magnetising field of *H *= 2000 A/m is applied for *t *= 1 s. 450 *μ*s after switching off the field, a highly sensitive low-*T*_C_)-SQUID (Superconducting QUantum Interference Device) sensor measures the magnetic induction *B*(*t*) at a distance of 10 mm above the sample. The measurement time window is 450 *μ*s ≤ *t *≤ 0.45 s. The output signals of SQUID sensors contain an unknown offset value *B*_offset_. For visual comparison purposes, the offset of each measured relaxation curve *B*(*t*) was adjusted so that all relaxation curves coincide at the end of the measurement.

Note, that the relaxation signals can be measured also by high-*T*_C_-SQUIDs [10] or by fluxgate magnetometers [21]. In the latter case the sensitivity is about 2 orders of magnitude lower, but there is no need for cost intensive cooling procedures. The relaxation behavior of MNP can also be measured with magneto-optical methods as described for example in [22].

The total magnetic moment of the sample is the sum of the magnetic moments of all MNPs. The magnetic moment of a single MNP can reorient within the MNP itself by the Néel relaxation mechanism with the time constant *τ*_N _= *τ*_0 _exp{*KV*/*k*_B_*T*} [23], where *τ*_0 _= 10^-10 ^s [24] and *K *and *V *are the anisotropy constant and the volume of the MNP core, respectively. If the MNP is not immobilised, the magnetic moment can relax *additionally *via the Brownian relaxation mechanism with the time constant

$$\tau_{\text{B}}=\pi \;\eta d_{\text{hyd}}^{3}/(2\;k_{\text{B}}T).$$

The hydrodynamic diameter of the whole particle (including the shell) is denoted by *d*_hyd _and *η *is the dynamic viscosity of the fluid suspension being 10^-3 ^Pa s for water. *k*_B _and *T *are the Boltzmann constant and the temperature, respectively. Note that the effective relaxation time *τ*_eff _of unbound MNP is always shorter than that of immobilised MNP and obeys the equation $\tau_{\text{eff}}^{-1}=\tau_{\text{N}}^{-1}+\tau_{\text{B}}^{-1}$.

The measured relaxation curve *B*(*t*) can be described as a superposition of the relaxation curves of bound, *B*_b_(*t*), and unbound probes, *B*_ub_(*t*). Thus, we estimated the fraction *β *of bound MNP by fitting the model [14]

*B*(*t*) = *β B*_b _(*t*) + (1 - *β*) *B*_ub _(*t*) + *B*_offset_

to the measured relaxation curves. The difference between relaxation times of bound and unbound MNP crucially determines the accuracy by which the corresponding signals can be separated.

If the molecules to be quantified are comparatively small in comparison to the probes, i.e. the MNP-labelled detection molecules, then the binding of the analytes to the probes does not lead to a significant slowing down of the Brownian motion. But this slowing down is necessary for a sensitive quantification of the analyte. Therefore, in our preparation the analyte was coupled to agarose beads being much larger (*d *≈ 5 *μ*m) than the probes. After the probes became bound to these analyte-beads via the detection molecules, the Brownian relaxation time of the probe-bead conjugates attains values of *τ*_B _≈ 400 s according to (1). This is orders of magnitude more than *τ*_B _≈ 2 ms which is measured for free probes (see below). Because this time is far beyond the upper limit of our observation time *t*_m,max _= 0.45 s, the MNP appear immobilized in our measurement. Thus, for the estimation of the amount of bound MNP by means of (2) we can use the relaxation curve of freeze dried MNP as the reference data for *B*_b_(*t*).

### Binding kinetics

Generally, one is interested in the binding kinetics between analyte and detection molecules. However, in marker based assays, the kinetics of the detection molecules including the marker is measured. (Note, that there are other assays working without markers e.g. SPR.) Caused by the markedly reduced diffusion of labelled streptavidin due to the large size of the MNP-marker in the present case, we expect a slower reaction than for assays which use smaller markers, e.g. dyes. This reduction has to be accounted for in order to compare our results with those of other methods.

In the following we describe the binding reaction between the probes and the binding sites (the analytes) of the agarose beads which is realised by the coupling between detection molecules and the analyte molecules. The reaction between probes and binding sites, denoted by P and B respectively, writes

$$\text{P}+\text{B}\overset{k_{\text{a},\text{P}}}{\underset{k_{\text{d},\text{P}}}{⇌}}\text{PB}.$$

The association rate and the dissociation rate constants *k*_a,P _and *k*_d,P_, respectively, describe the kinetics of the binding of the probes (MNP-detection molecule complex). The symbols *k*_a _and *k*_d _stands for the rate constants of the naked molecules alone.

According to our experimental conditions, we treat the case where the concentration of the probes exceeds that of the targets, i.e. the concentration of the agarose beads (see Table 3). Then, the increase in number of reaction products (PB), d*n*_PB_, in a small time interval d*t *is proportional to the actual concentration *c*_P _of unbound probes (P) in the vicinity of the targets and the maximal number of bounds *n*_PB,max _which can be realised:

**Table 3:** Concentrations and size parameters of the probes and biotin-agarose beads.

			Bead concentration	
Samples	Volume concentration of magnetite *c*_*V*_	Mean bead diameter $\bar{d}$ nm	Stock susp. *c *pmol/1	Meas. samples *c *pmol/1
biotin-agarose		5000	0.85	0.19
MNP*SAV	1.5·10^-4^	50	58721	6525/1958
MNP*AB	1.7·10^-3^	50	680190	22670/2267

d*n*_PB _= *k*_a,P _*c*_P _*n*_PB,max _d*t*.

In the same time interval, according to (3), the same amount of PB-complexes,

d*n*_PB _= *k*_d,P _*n*_PB _d*t*,

dissociates. Assuming that the kinetics is purely diffusion driven (far from mass transport regime), the concentration of the probes is homogeneous within the sample volume and writes

*c*_P _(*t*) = *c*_P,0 _- *c*_PB _(*t*).

In the given situation, the total number of the binding sites B of the *multivalent *target entities (agarose beads) exceeds the number of the probes in the samples strongly (see methodical section). Therefore, the maximal number of bounds *n*_PB,max _which can be realised is equal to the initial number of probes *n*_P,0_. Thus, the combination of (4), (5) and (6) yields the differential equation describing the evolution of the concentration *c*_PB _= *n*_PB_/*V *(*V*-sample volume) to be

$$\frac{\text{d}c_{\text{PB}}(t)}{\text{d}t}=k_{\text{a},\text{P}}c_{\text{P},0}(c_{\text{P},0}-c_{\text{PB}}(t))-k_{\text{d},\text{P}}c_{\text{PB}}(t).$$

The solution of (7) writes

$$c_{\text{PB}}(t)=\frac{k_{\text{a},\text{P}}c_{\text{P},0}^{2}}{k_{\text{a},\text{P}}c_{\text{P},0}+k_{\text{d},\text{P}}}.$$

·(1 - exp{-(*k*_a,P _*c*_P,0 _+ *k*_d,P_)*t*}).

If the dissociation rate *k*_d,P _is much less then the binding rate *k*_a,P _*c*_P,0_, the solution of (7) simplifies to

*c*_PB _(*t*) = *c*_P,0 _(1 - exp{-*k*_a,P _*c*_P,0 _*t*}).

In this case, the association rate constant can be calculated straight forward to be

*k*_a,P _= (*c*_P,0 _*t*_b_)^-1^

where *t*_b _is the binding time at which the (1-1/e) part of the number of bindings at saturation is realised.

Eventually, we are interested in the kinetics of the unlabelled biomolecules. As mentioned above, the association rate constant of the probes is expected to be smaller in comparison to that of unlabelled detection molecules due to the large size of the marker. If we want to infer from the association rate constants of the probes to that of the naked detection molecules, we have to take into account their different diffusion constants *D *∝ $r_{\text{hyd}}^{-1}$ where *r*_hyd _is the hydrodynamic radius. Thus, the association rate constant of unlabeled molecules calculates

*k*_a _= *κ k*_a,P_,

where *κ *= *r*_hyd,P_/*t*_hyd_. In this approximation the reduction of the rotational diffusion coefficient *D*_rot _∝ $r_{\text{hyd}}^{-3}$ was not taken into account. This latter effect should be strong only if the surface of MNP is sparsely covered by binding molecules.

### Experimental

We investigated the binding between biotin as the analyte and the detection biomolecules streptavidin and anti-biotin antibody using a bead based MARIA. As described above, the measurement signal is generated by immobilization of the MNP being the labels of streptavidin (SAV) and anti-biotin antibody (AB) by their binding to the biotin which is linked to agarose beads.

#### Materials

Biotin-agarose was purchased from Sigma-Aldrich (Germany), order No: B 0519 and has a binding capacity of 20–30 mg/ml avidin, i.e. 0.3–0.5 mmol/l. The diameter of the beads is about 5 *μ*m. Streptavidin and anti-biotin antibody, both labelled by MNP, were purchased from Miltenyi Biotec (Bergisch-Gladbach, Germany). We will refer to them further as MNP*SAV (Streptavidin-linked MicroBeads, order No: 130-048-102) and MNP*AB (Antibiotin-antibody-linked MicroBeads, order No: 130-090-485), respectively (Table 3). For the binding experiments in different media we used an other sample of MNP-labelled streptavidin, referred to as MNP*SAV2 (Streptavidin-linked MicroBeads, order No: 130-074-101).

The suspension media for the binding reactions are phosphate buffered saline (PBS) and human serum (Sigma-Aldrich, order No: B 0519) for the measurements of the influence of suspension medium.

#### Binding experiments

The binding experiments were performed as follows: The biotin-agarose beads were washed 3 times with PBS to eliminate residues of free biotin which would saturate the MNP*SAV and MNP*AB probes. We diluted the stock suspension of biotin-agarose by a factor of 3 with PBS. The MNP*SAV were diluted with PBS by factors of 3 and 10; MNP*AB were diluted by factors of 10 and 100. For the MARIA-measurement we filled 100 *μ*l biotin-agarose in a polystyrene-well (BreakApart-wells, Nunc GmbH & Co.KG, Germany). Then, we added 50 *μ*l of the MNP*SAV or MNP*AB suspension. The final concentrations of MNP*SAV, MNP*AB and agarose beads in the 150 *μ*l measurement volume are listed in Table 3. After gently mixing we measured the magnetic relaxation.

Because the agarose have slightly higher density (*ρ *= 1.27 g/cm^3^) than water, the beads tend to sediment. However, because one measurement is done after about 5 s, this sedimentation does not effect the measurement significantly.

For the control experiment we incubated MNP*SAV and MNP*AB suspensions with free biotin with a concentration of *c *≈ 30 *μ*mol/l for about 3 hours at room temperature (22°C). The high biotin excess should saturate the biotin binding sites of streptavidin and antibiotin-antibody. With this saturated suspensions we repeated the binding experiments as before. In order to measure the binding kinetics, we estimated the fraction of bound MNP*SAV and MNP*AB at different times *t*_I _after the incubation was launched by fitting (2) to the relaxation curves.

Because the size of the beads reaches the *μ*m-range and they have a slightly higher density than water, the beads tend to sediment due to gravity forces. Therefore, we investigated, how the treatment of the samples during the binding reaction determines the kinetics. In the first set of experiments, the samples were in rest between each single relaxation measurement. Only before each measurement, the samples were shaken once to obtain a homogeneous distribution of the agarose beads, because the measurement signal depends on the sample detector spacing. In the second set of experiments, the samples were rotated slowly between the measurements, providing a continuous stirring of the agarose.

In order to examine the influence of the suspension medium on the binding reaction, we repeated the binding experiments as described above substituting PBS by human serum.

Because of the sedimentation tendency of the agarose beads, the homogeneity of the sample gets lost after a couple of seconds. But due to the short measurement time of only 5 seconds, this has no impact to the given results. In the case of longer measurement cycles, e.g. short time kinetics measurement, there is a need of sample stirring preventing the sedimentation of the beads.

## Appendix

We describe the kinetics for the binding of magnetic probes to beads, *β*(*t*_I_), by a model, developed earlier for analysing the kinetics of the binding of electrically charged MNP to oppositely charged latex beads [14]. The model bases on the diffusion driven collision theory of Smoluchowski [25]. In this model only bindings between elements of *different *components, namely the probes denoted by P, and binding sites of the beads, denoted by B, are taken into account.

The number density *c*_P _(*t*) of probes, which are unbound at the time *t*, obeys the differential equation [14]:

$$\frac{\text{d}c_{\text{P}}(t)}{\text{d}t}=-\alpha_{\text{i}}D_{\text{P}}c_{\text{B}}(t)c_{\text{P}}(t),$$

where *α*_i _is the collision parameter and *D*_P _is the diffusion constant of the probes. The total concentration of binding sites *c*_B _(0) which are accessible by ligands is related to that of the agarose beads by *c*_B _(0) = $\alpha_{\text{F}}\pi d_{\text{A}}^{2}c_{\text{A}}$ where *d*_A _and *c*_A _are the diameter and the concentration of the agarose beads, respectively. The dimensionless parameter *α*_F _represents the fraction of the surface area of the beads which can be covered by specific binding probes at most.

As the binding goes on, the surface of the agarose beads becomes more and more covered by the probes and the concentration of unoccupied biotin targets *c*_B _(*t*) decreases. The solution of (13) is [14]

$$\frac{c_{\text{P}}(t)}{c_{\text{P},0}}=\frac{c_{\text{P},0}A_{\text{C}}-\alpha_{\text{F}}c_{\text{A}}q}{c_{\text{P},0}A_{\text{C}}-\alpha_{\text{F}}c_{\text{A}}q\;f(t)}$$

with

$$f(t)=\exp\left\{-\frac{\alpha_{\text{B}}}{\alpha_{\text{F}}}(c_{\text{P},0}A_{\text{C}}-\alpha_{\text{F}}c_{\text{A}}q)\;2\frac{D_{\text{P}}+D_{\text{A}}}{d_{\text{P}}+d_{\text{A}}}t\right\}$$

and the cross section area of the probes, $A_{\text{C}}=\frac{\pi }{4}d_{\text{P}}^{2}$, and the cross section of collision between probes and agarose beads, *q *= *π *(*d*_P _+ *d*_A_)^2^, where *d*_P _is the overall diameter of the probes. *D*_A _is the diffusion constant of the agarose beads. The binding parameter *α*_B _describes the probability that a collision between probes and agarose results in a binding.

Equation 14 describes the disappearance of unbound probes. Thus, the amount of bound probes *β *(see equation 2) writes

$$\beta (t)=\beta_{\max}\left(1-\frac{c_{\text{P}}(t)}{c_{\text{P},0}}\right)$$

*β*(*t*)/*β*_max _corresponds to *c*_PB _(*t*)/*c*_P,0 _in equation 10. *β*_max _is the maximal fraction of probes which can bound to agarose beads. It is determined by the fraction of MNP which are grafted by detection molecules in the proper orientation making a binding possibly. If the dissociation rate constant *k*_d _is not negligibly, *β*_max _is also determined by the position of the equilibrium of the reaction.

## Authors' contributions

Authors DE and CB were responsible for concept and the planning of experiments. Experiments, data analysis were performed by DE and FW. Discussion of the results and creating the final manuscript were done by DE, US, FW and LT. All authors have read and approved the final manuscript.
